# Gigapixel big data movies provide cost‐effective seascape scale direct measurements of open‐access coastal human use such as recreational fisheries

**DOI:** 10.1002/ece3.4301

**Published:** 2018-08-27

**Authors:** David J. H. Flynn, Tim P. Lynch, Neville S. Barrett, Lincoln S. C. Wong, Carlie Devine, David Hughes

**Affiliations:** ^1^ The Commonwealth Scientific and Industrial Research Organisation (CSIRO) Oceans & Atmosphere Hobart TAS Australia; ^2^ The Institute for Marine and Antarctic Studies (IMAS) University of Tasmania Hobart TAS Australia

**Keywords:** big data, coastal, CSIRO Ruggerdised Autonomous Gigapixel System, GigaPan, open‐access fisheries, photomosaic, remote sensing, southern bluefin tuna, time interval count, time‐lapse

## Abstract

Collecting data on unlicensed open‐access coastal activities, such as some types of recreational fishing, has often relied on telephone interviews selected from landline directories. However, this approach is becoming obsolete due to changes in communication technology such as a switch to unlisted mobile phones. Other methods, such as boat ramp interviews, are often impractical due to high labor cost. We trialed an autonomous, ultra‐high‐resolution photosampling method as a cost effect solution for direct measurements of a recreational fishery. Our sequential photosampling was batched processed using a novel software application to produce “big data” time series movies from a spatial subset of the fishery, and we validated this with a regional bus‐route survey and interviews with participants at access points. We also compared labor costs between these two methods. Most trailer boat users were recreational fishers targeting tuna spp. Our camera system closely matched trends in temporal variation from the larger scale regional survey, but as the camera data were at much higher frequency, we could additionally describe strong, daily variability in effort. Peaks were normally associated with weekends, but consecutive weekend tuna fishing competitions led to an anomaly of high effort across the normal weekday lulls. By reducing field time and batch processing imagery, Monthly labor costs for the camera sampling were a quarter of the bus‐route survey; and individual camera samples cost 2.5% of bus route samples to obtain. Gigapixel panoramic camera observations of fishing were representative of the temporal variability of regional fishing effort and could be used to develop a cost‐efficient index. High‐frequency sampling had the added benefit of being more likely to detect abnormal patterns of use. Combinations of remote sensing and on‐site interviews may provide a solution to describing highly variable effort in recreational fisheries while also validating activity and catch.

## 1 INTRODUCTION

Worldwide, coastal zones are some of the most heavily impacted parts of the ocean by people, both in terms of activity types and participation rates (Halpern et al., 2008). A particular focus of research on coastal use has been recreational fishing as this popular activity can significantly affect fish and invertebrate stocks (Arlinghaus, 2006; Cooke & Cowx, 2004; Lewin, Arlinghaus, & Mehner, 2006; McPhee, Leadbitter, & Skilleter, 2002), as for many species, harvest can exceed the take of the commercial fishery (Giri & Hall, 2015; Lyle, Stark, & Tracey, 2014; Zischke, Griffiths, & Tibbetts, 2012).

Unlike commercial fisheries, which are commonly documented by operators logging their catch and effort, assessments of open‐access, nonreporting activities, such as recreational fisheries, require sampling. This can be both difficult and expensive due to their often large spatial extent, high temporal variation and, for the fisheries agencies undertaking the work, which often requires weekend and holiday surveying, high labor costs (Ma et al., 2018; Pollock, Jones, & Brown, 1994; Rocklin, Levrel, Drogou, Herfaut, & Veron, 2014; Venturelli, Hyder, & Skov, 2017). Due to these issues, assessments have typically relied upon indirect data collection from off‐site telephone surveys based on data frames developed around regional ‘white pages’ or landline telephone directories, which traditionally have had high response rates and easily scalable sample sizes (Moore et al., 2015; Pollock et al., 1994). Such approaches may now risk undersampling active fishers because of demographically bias and overall decreased landline phone use; unlisted and nonregional coded mobile phones and low response success from voice calls to mobile phones (Badcock et al., 2016; Blumberg & Luke, 2009; Teixeira, Zischke, & Webley, 2016).

Other forms of recreational fishery assessments employ direct on‐site survey methods such as bus‐route surveys, which use clerks traveling around a fishery—following randomly selected predetermined schedules—interviewing and observing fishers to track fishing effort, catch, and release (McGlennon & Kinloch, 1997; Pollock et al., 1994). On‐site methods that cover broad geographic areas, however, are seldom performed due to high labor costs (Jones & Pollock, 2012).

Given these limitations, remote sensing tools and technologies such as autonomous photography are an emerging field, offering direct monitoring as an alternative or supplement to on‐site interviews (Hartill, Payne, Rush, & Bian, 2016; Keller, Steffe, Lowry, Murphy, & Suthers, 2016; Parnell, Dayton, Fisher, Loarie, & Darrow, 2010; Powers & Anson, 2016; van Poorten, Carruthers, Ward, & Varkey, 2015; Wood, Lynch, Devine, Keller, & Figueira, 2016). One such system is the CSIRO Ruggedised Autonomous Gigapixel System (CRAGS), which is a programmable, weatherproof, camera trap that provides ultra‐high resolution, time‐lapse, and high‐frequency panoramic images. The CRAGS utilizes modified commercially available hardware and software known as a GigaPan^®^ (http://gigapan.com/) to capture photographic samples with such high pixel density (gigapans) that wide fields of view and associated objects of interests can be examined closely without the loss of broader environmental context at landscape or seascape scales (Lynch, Alderman, & Hobday, 2015) (Supporting Information Appendix S1).

This robotic camera system allows for much broader scaled photographic assessments than simple camera traps by autonomously taking, and then stitching together, multiple telephoto megapixel images into high‐resolution gigapixel panorama. The resulting tiled image allows fully zoomable viewing for either multiple small targets, such as Albatross nests across a colony (Lynch et al., 2015) or more widely spaced larger targets, such as identifying commercial vs. recreational vessels around an offshore artificial reef (Wood et al., 2016). The benefit of the system compared to simpler camera traps is being able to observe, in high detail from the one image stream, many objects simultaneously across landscape or seascape scales. With so much information, data handling can become overwhelming so a batch processing method called Gigapan Time machine has been developed. Originally used for viewing cosmological simulations (Yu et al., 2011), Time Machine produces a video stream that allows viewers to fluidly explore giga to tetra pixel‐scaled videos across both space and time.

**Figure 1:**
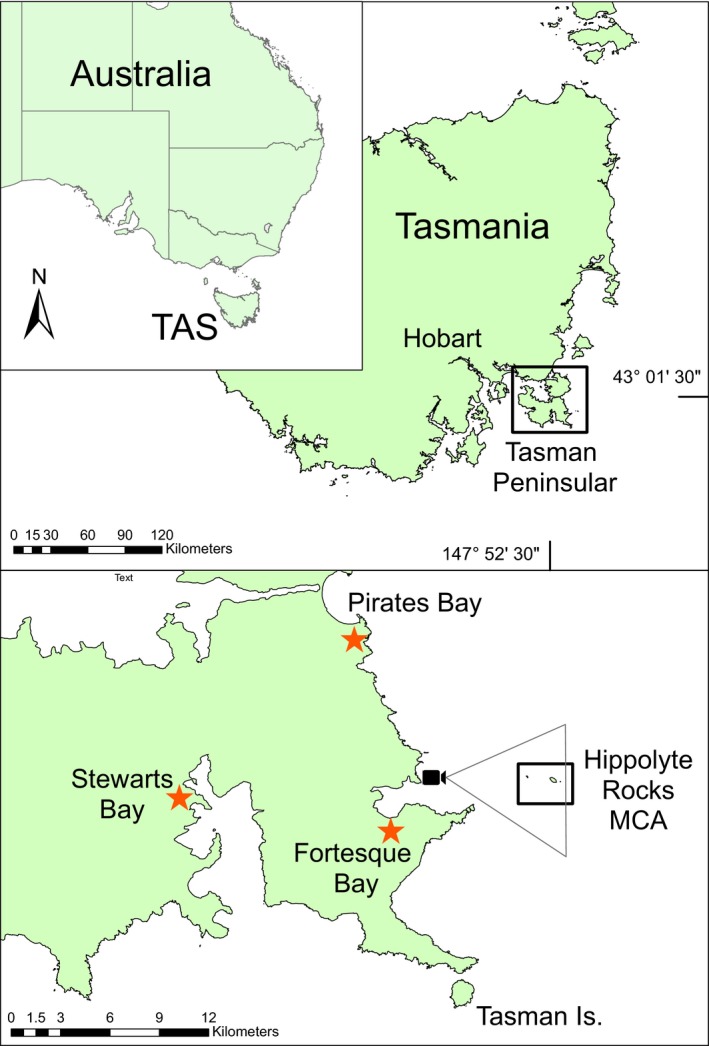
The Tasman Peninsula in SE Tasmania. Bus‐route survey sampling occurred at ramps 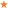 located at Pirates, Fortescue and Stuarts Bay. The CRAGS location is shown as a camera icon and the area observed by the camera, which includes the Hippolyte Rocks Marine Conservation Area (HRMCA), is described as a triangle

In Australia, annual recreational fishing participation has been estimated at 19.5% (Henry & Lyle, 2003), which is well above the global average of around 10% (Arlinghaus, Tillner, & Bork, 2015). Around the Australian island state of Tasmania, the annual participation rate is 29.3%, which well exceeds the national average. The Tasman Peninsula in Tasmania's southeast (Figure 1) is a popular fishing region near to the largest city and state capital of Hobart, where a steep bathymetric profile and migratory game‐fish pathway overlap, providing coastal access to normally offshore fishing opportunities to recreational fisher in trailer boats. Pelagic game fish such as southern bluefin tuna (*Thunnus maccoyii*) are targeted in late Austral Summer and Autumn (Morton & Lyle, 2004), but as the fishery is unlicensed and episodic, trends in effort and catch are poorly understood (Lowry & Murphy, 2003; Moore et al., 2015). This particular recreational fishery is also of more general interest as the targets are commercially important species, which include those with internationally negotiated quotas (Pons et al., 2017; Zischke et al., 2012). Recreational fishers are also often associated with other users of the marine environment (Farr, Stoeckl, & Sutton, 2014; Kearney, 2002) or in this case tourists enjoying the local national parks. Hence, high use sites may be important to monitor not only for fisheries and ecological reasons but for managing social values, conflict resolution and planning (Alessa, Kliskey, & Brown, 2008; Lynch et al., 2004).

We trailed our CRAGS method to see whether we could develop a high‐frequency time series of temporal trends of observed trailer boat fishing effort at an offshore concentration point for the recreational game fishery. We also tested both the suitability and labor cost‐effectiveness of the hardware and a novel application of Time Machine batch processing software to automatically produce “big data” interactive movies made from our gigapixel panoramas. As the peninsula is isolated and serviced by only a limited number of boat ramps, we aimed to validate the representativeness of the temporal trends we observed with CRAGS by correlating to match samples obtained from a simultaneous, regionally scaled bus‐route trailer boat survey at the three ramps on the peninsular suitable for launching large trailer boats. We also undertook on‐ground interviews with trailer boat users to ground truth activity type (fishing or nonfishing) and target species. By undertaking both camera and boat ramp surveys, our over‐overarching aim was to investigate how remote imagery may replace, compliment, or optimize conventional on‐site approaches for recreational fishing.

## 2 MATERIALS AND METHODS

**Figure 2:**
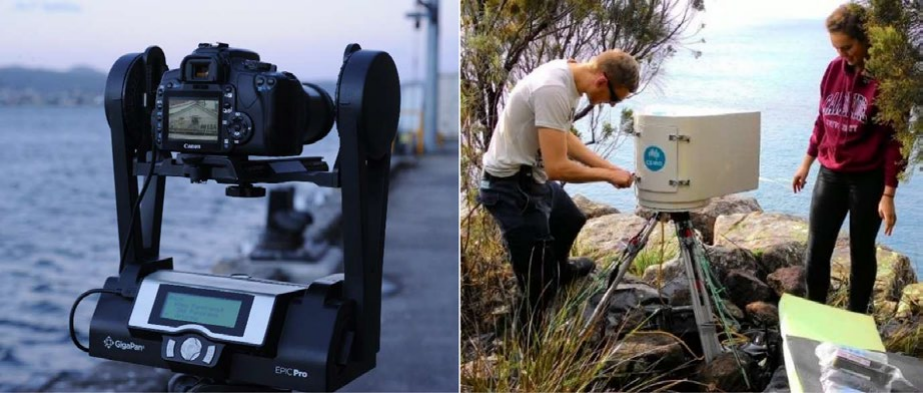
An unmodified GigaPan Epic PRO unit (left) and a CRAGS deployed at Waterfall Bay, SE Tasmania (right)

We deployed the CRAGS between 1 May 2015 and 30 June 2015 to assess trailer boats offshore from water adjacent to the Tasman Peninsula (Figure 1). This period was chosen as it corresponds with the main tuna fishing season. For large trailer boats (>5 m length), local access to the fishery is limited to three boat ramps (Figure 1), although the area may also be visited by larger vessels steaming from marinas and anchorages further north (Morton & Lyle, 2004), but for the purposes of our study, we excluded these relatively rare larger vessels. We focused our CRAGS survey at a known game fishing concentration, the Hippolyte Rock Marine Conservation Area (HRMCA), which are a group of small islands about 6‐km offshore (Figures 1 and 2) around which recreational fishing is permitted. While the CRAGS deployment was marginally closer to the Fortescue Bay Ramp, we commonly observe boats approaching the HRMCA from directions that corresponded to the location of all three ramps. We fixed the CRAGS to a clifftop at Waterfall Bay which overlooks the site from very high sea cliffs (~250 m), with the camera focusing on the western side of HRMCA (Figure 1). For each sample, CRAGS captured a series of 68 photographs through a telephoto lens, which when stitched together provide a high‐resolution panoramic image with a wide field of view (~140°) (Supporting Information Appendix S1). The size of area surveyed by CRAGS, compared to the regional area accessible by trailer boats, was estimated using google earth polygons and the University of New Hampshire KML extension tool—https://extension.unh.edu/kmlTools/index.cfm.

The time of the initial CRAGS panorama sample was randomly selected. Samples were then collected sequentially throughout daylight hours (06:00–18:00). As less recreational fishing for tuna occurred at night, no samples were taken, with the CRAGS programmed to “blank” these hours from the sampling schedule (Supporting Information Appendix S1). Photographic sessions were programmed to be separated by a recurring 93‐min gap, thus producing a forward shift in sample capture time and minimizing potential autocorrelations between boating activities and sampling times. Photographs were batch processed to both form panoramic images for each sample and for compiling into an interactive time‐lapse movie using CREATE Lab's Time Machine software (CREATE Lab^®^ Pittsburg, PA, USA).

**Figure 3:**
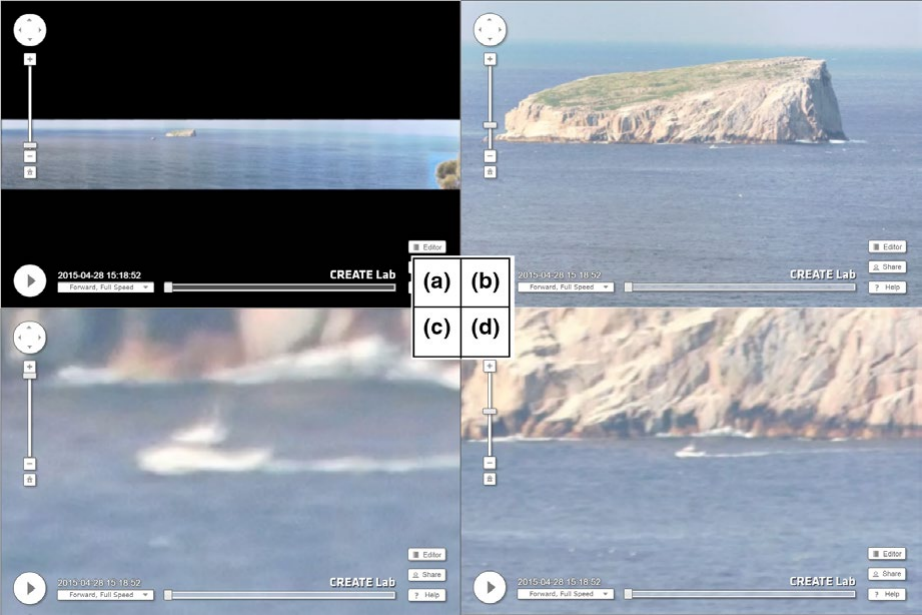
Screen capture outputs from the Time Machine interactive data viewer showing: (a). no zoom and panoramic extent of the Hippolyte Rock Marine Conservation Area (HRMCA), (b) = 1/3 zoom, main Hippolyte rocks ~6 km from camera, (c) = 1/2 zoom of a large trailer boat, (d) = full zoom

Time Machine movies are fully interactive and are able to be zoomed into, paused, and played at various speeds and are also time and date‐stamped (Figure 3). Using this interactive movie software, each GigaPan movie was systematically paused at each sample, fully zoomed, and scanned left to right, top to bottom, for objects in the seascape by the lead author (Figure 3). Vessel counts were recorded for each sample with objects larger than ~10 m in length (not trailer boats) or requiring longer than 10 seconds to distinguish (e.g., low resolution and image artifact) being discounted. Samples were categorized into two strata: weekdays and weekends/public holidays using the time and date stamp provided on each GigaPan scene (Figure 3).

The count of boats for each sample was then extrapolated into an estimate of daily average boating effort (hours ± *SE*) using the instantaneous count method (Equation (1)).

(1) $${\hat{e}}_{i}={\overset{¯}{I}}_{i}\times T$$

where ${\hat{e}}_{i}$ is the extrapolated boating hours for the *i*th day, ${\overset{¯}{I}}_{i}$ is the average number of trailer boats observed across the instantaneous samples for day *i*, and *T* is the daily sampling period (daylight hours). As bad weather can be a major factor affecting the decision to boat or fish (Forbes, Tracey, & Lyle, 2009), boating effort was assumed to be 0 when adverse weather made observations of boats impossible.

Next, total effort for each day‐type strata (${\hat{E}}_{\mathrm{jk}}$) within sampling months (*k*) was calculated to estimate the monthly effort (Equation (3)). As CRAGS allowed for continuous sampling throughout the survey period, the sampling probability (*π*_*k*_) was set to 1.

(2) $${\hat{E}}_{\mathrm{jk}}=\sum_{i=1}^{n}\left(\frac{{\hat{e}}_{i}}{\pi_{k}}\right)$$

Standard error and effort variance for monthly estimates were calculated (Equation (3)) according to Pollock et al. (1994) for strata.

(3) $$SE_{\mathrm{jk}}=\sqrt{\frac{\frac{1}{n_{j}-1}\sum_{i=1}^{n}{\left({\hat{e}}_{i}-{\overset{¯}{e}}_{i}\right)}^{2}}{n_{j}}\times {\left(N_{\mathrm{jk}}\right)}^{2}}$$

where *n*_*j*_ is the number of sampled days of strata *j*;* N*_*jk*_ is the total number of days of strata *j* in month *k*.

As variation for each strata was calculated separately, we used a sum of squared standard errors approach (Equation (4)) to estimate the total monthly variation.

(4) $$SE_{k}=\sqrt{SE_{1k}^{2}+SE_{2k}^{2}\ldots +SE_{\mathrm{jk}}^{2}}$$

As samples were obtained in a serial and continuous fashion, a time series model using rolling averages was also applied to the data (Diggle, 1995) (Equation (5)).

(5) $${\text{sm}}_{t}=\frac{{\hat{e}}_{t-1}+{\hat{e}}_{t}+{\hat{e}}_{t+1}}{3}$$

where sm_*t*_ is the smoothed average of extrapolated effort $\hat{e}$ per sample *t* which is weighted across three samples, each separated by 93 min. This provided a smoothed average within days.

We also conducted a bus‐route style survey to estimate fishing effort from trailer boats for the region (Pollock et al., 1994; Robson & Jones, 1989). While CRAGS samples were collected throughout May and June, the comparable bus‐route survey was only conducted in May due to logistical constraints. We surveyed the three major boat ramps in the region at Pirates Bay; Fortescue Bay; and Stewarts Bay between 3 May 2015 and 30 May 2015 (Figure 1).

A total of 20 bus‐route samples were scheduled. We undertook random stratified sampling with day type divided into weekdays or weekend/public holidays. To reflect expected higher recreational effort during weekends and public holidays (McCluskey & Lewison, 2008), the sampling probability (*π*_*k*_) was weighted toward this strata over weekdays at 12 to eight samples (Supporting Information Appendix S2). We stratified the sample time into morning (a.m., 06:00–11:59) and afternoon (p.m., 12:00–18:00), with equal probabilities of sampling. To minimize temporal autocorrelation, the starting location and travel direction of the route were also randomly selected without replacement. Equal sampling weight was provided to each ramp due to lack of prior knowledge of use rates.

At each boat ramp, surveys were conducted by first counting parked boat trailers, then by monitoring vessel entry and exits during a 1‐hr sampling period (Kinloch, McGlennon, Nicoll, & Pike, 1997; Pollock et al., 1994). The 1‐hr period was chosen due to travel time and distance between sites, allowing for a bus‐route sample to be completed for all ramps within the temporal strata (AM or PM) of the daily sampling frame. Interviews were conducted with all parties launching and retrieving vessels during the clerk's wait time, determining party size, target species/group, and activity (i.e., fishing or not) for estimates of fishing effort. This well‐established procedure produced a monthly extrapolated estimation of boat effort in standardized units of effort hours ± *SE*, which could then be adjusted to fishing effort via the proportion of interviewees activities (Pollock et al., 1994; Robson & Jones, 1989) (Supporting Information Appendix S3). Party size between ramps was tested for any difference using a one factor ANOVA.

The bus‐route survey hence provides an estimation of all fishing effort from trailer boats around this isolated peninsular. The CRAGS data are thus a high‐frequency spatial subset of the entire trailer boat fishing effort for the period that was extrapolated by the regional survey. This allowed us to both test the relative use of the HRMCA compared to wider peninsular use and the ability of our remote observations with our CRAGs unit of an actual subset of the fishery to track larger temporal patterns of peninsular wide effort.

To validate the representativeness of the CRAGS high‐frequency tracking of effort metrics over time, we undertook a Spearman rank correlation between time‐matched effort estimations by CRAGS and the regional bus‐route survey. For graphing, bus‐route extrapolation estimates were overlaid onto extrapolations from daily averages of boat fishing effort from CRAGS. We also carefully logged all time spent on the project to compare total labor costs between CRAGS and the bus route for both data collection and processing to determine effort across the comparative month as well as for cost per sample.

## 3 RESULTS

The CRAGS operated successfully and continuously throughout the study, capturing 5.45 ± 1.98 *SE* (May) and 4.83 ± 0.88 *SE* (June) panoramas per day, making a total of 314 samples. We made 895 observations of boats but 51 samples across 21 days returned zero counts of boats (17% of total samples). These were concentrated on weekdays—with 44 zero‐count samples across 18 days—while on weekends and public holiday there were only seven samples across 3 days with no activity. Inclement weather rendered 27 samples unusable. The images and batch‐processed Time Machine movies consumed 427.4 GB of memory.

**Figure 4:**
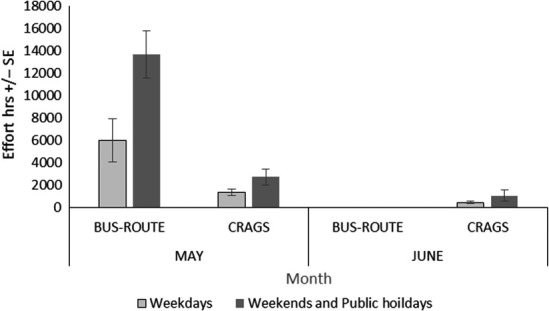
Extrapolated monthly trailer boat effort (hours ± *SE*) for the entire Tasman Peninsula, assessed by a bus‐route survey (May) and for the subset of the area around the HRMCA sampled with CRAGS (May and June)

Based on boat ramp interviews the average trailer boat party size ($\hat{P}$) was 2.87 people per vessel throughout May (±0.133 *SE*,* n* = 84), and party size between sites showed no significant difference (*df* = 2, *F *=* *3.02, *p *=* *0.68). The average monthly boat fishing effort (hours) extrapolated for our CRAGS observations of HRMCA was 5,629 hr (±711 *SE*), with 4,096 hr (±607 *SE*) in May and 1,534 hr (±370 *SE*) in June (Figure 4)—this could be extrapolated to fisher hours by multiplying by the average party size but as no interviews were conducted in June, we left both the CRAGS and bus route extrapolations as boat fishing effort only.

The total subset area observed by CRAGS was 9.6 km^2^ which compared to 318 km^2^ that was easily available to trailer boats around the Tasman Peninsular, which corresponds to around 3% of the total area. Compared to the total fishing effort for May, extrapolated from the regional bus‐route survey, the relatively small area around the HRMCA (~3%) received a large amount of the fishing effort (~23%).

**Figure 5:**
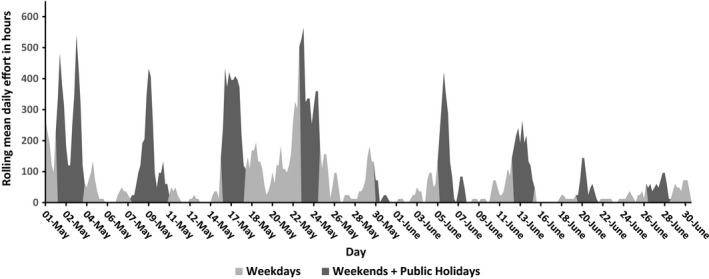
High‐frequency, interdaily, extrapolated CRAGS estimations of boat effort hours around the (HRMCA)

The high frequency of CRAGS sampling permitted the examination of fine‐scale inter‐daily temporal variation, which displayed a ragged sinusoid oscillation (Figure 5). Effort was generally low within the weekday strata and then increased rapidly to large peaks on weekends (Figure 5). One exception to this was between the 18th and 22nd May, which had more effort on weekdays than other periods (Figure 5). Bracketing this period were two weekends of fishing competitions, the “Tuna Club of Tasmanias’ Far South Classic” (16–17th May) and Rally #4 Northern invitational weekend rally (23rd May).

A total of 16 bus‐route samples were conducted; logistic failures led to the abandonment of four (20%) of the planned samples. Regional trailer boat effort from around the Tasman Peninsular was estimated to be 19,650 hr (±2,858 *SE*) in May (Figure 4). No trailers at individual ramps were recorded four times, with all of these occurring on weekdays. The total number of trailers sighted per ramp ranged from 0 to 16 during weekdays and 1–54 for weekends, with the highest number recorded on 16 May 2015 at Pirates Bay. During high‐intensity days (particularly weekends/holidays mornings), there were often large overflows from ramp car parks, with parked cars and boat trailers extending as much as one kilometer from the boat ramp.

During the bus‐route survey, 84 interviews were conducted. The majority of mariners (90.5% *n* = 96) indicated fishing was their primary activity (Table 1). Overall, 64.3% (*n* = 54) indicated their primary target were tuna, with 17.9% (*n* = 15) specifically targeting southern bluefin tuna (*Thunnus maccoyii*). Other common recreational targets included flathead (*Platycephalus* spp., 5.95%), Southern rock lobster (*Jasus edwardsii*, 10.7%), and squid/calamari (*Sepioteuthis australis* and *Nototodarus gouldi*, 4.76%) (Table 1).

**Table 1:** Interviewees reported primary target taxa or activity

Primary target taxa/species/activity	Interview responses	%
Tuna (unspecified)	37	44.0
Southern Bluefin Tuna	15	17.9
Albacore Tuna	2	2.38
All Tuna	54	64.3
Flathead	5	5.95
Southern rock lobster	9	10.7
Squid/calamari	4	4.76
Abalone	1	1.19
Small Pelagics	1	1.19
Snotty Trevalla	2	2.38
All nearshore species	22	26.2
Surfing	1	1.19
SCUBA	3	3.57
Demon Cave visitors	3	3.57
Pleasure Cruise	1	1.19
All nonextractive	8	9.52

*Note*

“Tuna” responses denote anglers targeting all species within the family *Thunnini*.

Based on the responses of activity type from interviews, total boat fishing effort in May was estimated to be 17,783 hr (±2,586 *SE*). As most nonfishing activities were near shore, we assumed trailer boating activity at HMRCA observed by CRAGS was all fishing.

**Figure 6:**
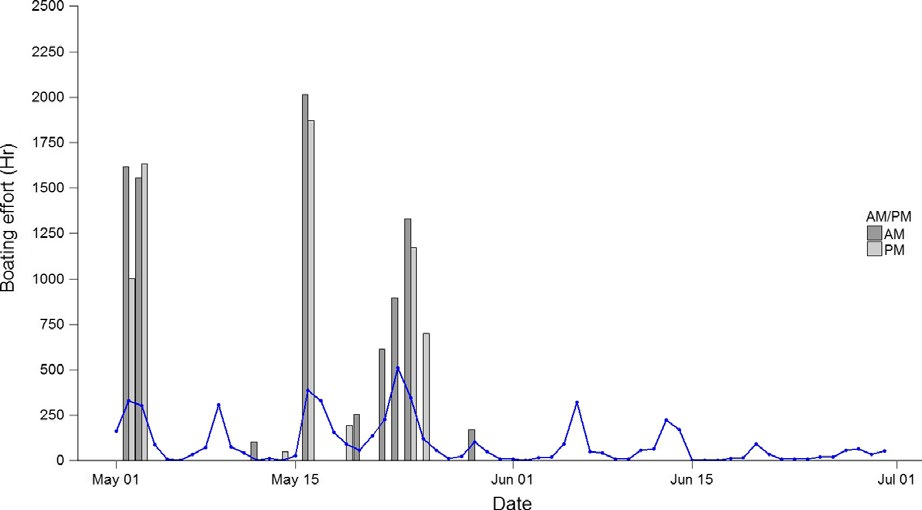
Daily boating effort (hours) extrapolated using bus‐route access point survey (bar) and CRAGS (line). Effort estimate from bus‐route survey was calculated from morning (a.m., dark gray) and afternoon (p.m., light gray) survey. CRAGS survey was conducted between May and June, while the bus‐route survey was only conducted in May

There were 16 direct comparison samples between the bus route and CRAGS methods, between the 2nd and the 29th of May. Samples consisted of 7 weekdays and 9 weekends/public holiday samples (Figure 6). In four cases where two bus‐route samples were taken on the same day (Supporting Information Appendix S2), they are combined to a daily estimation for tabulation (Table 2). The proportional difference between estimated efforts using the two methods ranged from 0.78‐ to 4.18‐fold difference; however, ranks estimates between CRAGS and bus‐route samples were strongly associated (*n* = 16, *ρ*(12) = 0.87, *p* = <0.01). Interdaily sampling strata (a.m./p.m.) were also examined separately for correlation, which remained strong for both a.m. (*n* = 9, *ρ*(7) = 0.80, *p* = <0.01) and p.m. (*n* = 7, *ρ*(5) = 0.89, *p* = <0.01).

**Table 2:** Ranking of effort for paired samples between CRAGS and the bus route

Date	CRAGS hours (rank)	Bus‐route hours (rank)	Δ Rank
2/05/2015	330 (9)	1,208 (10)	−1
3/05/2015	303 (8)	1,471 (11)	−3
12/05/2015	0 (1)	48 (2)	−1
14/05/2015	0 (1)	24 (1)	0
16/05/2015	387 (11)	1,794 (12)	−1
19/05/2015	93 (4)	89 (4)	0
20/05/2015	57 (3)	117 (5)	−2
22/05/2015	228 (7)	283 (6)	1
23/05/2015	513 (12)	414 (8)	4
24/05/2015	345 (10)	1,155 (9)	1
25/05/2015	122 (6)	323 (7)	−1
29/05/2015	102 (5)	79 (3)	1

For the comparative month, CRAGS took 169 samples compared to the bus routes’ 16 (Table 3). We had two field trip days for CRAGS with four people on the setup and three on the breakdown day, which resulted in seven full‐time equivalent (FTE) days of work. This compared to 16 field days for the bus‐route method, which included travel to the field site, for between two and three staff, which accumulated to 40 FTE days of work. Setting up our CRAGS automated stitching took one FTE day (though this is faster for experienced officers), while data extraction per image was estimated to take on average 7.5 min, compared to 1 min for entering the bus‐route data sheet. While data extraction for CRAGS took longer compared to the bus route—at 3.81 FTE days—total monthly labor for CRAGS took only a quarter of the time and on a per‐sample comparison was only 2.5% of the bus‐route labor cost.

**Table 3:** Full time equivalent (FTE) labor days for CRAGS and bus route surveys to estimate patterns of effort

	CRAGS	Bus route
Number of samples	169	16
Field days	2	16
Average people per field day	3.5	2.5
**Field FTE days subtotals**	**7**	**40**
Stitching setup FTE	1	0
Data extraction FTE (per sample)	0.017	0.002
**Subtotal data extraction FTE**	**3.81**	**0.04**
FTE days per sample	0.06	2.50
**Total FTE days**	**10.8**	**40.0**

## 4 DISCUSSION

Over our autumn sampling, most trailer boat owners accessing the waters around the Tasman Peninsula via the regional boating infrastructure were recreationally fishing, with fishers predominately targeting tuna. Trailer boat recreational fishers constitute a large tranche of coastal users in Australia (McPhee et al., 2002), and our results suggested that this region remains one where recreational fishing is concentrated (Morton & Lyle, 2004). Effort observed by CRAGS was a spatial subset of the general area easily accessed by trailer boats around the Tasman Peninsular, which we regionally assessed with our bus‐route roving survey. As CRAGS only observed ~3% of the total area but corresponded to ~23% of the monthly bus‐route boat effort, the Hippolyte Rocks Marine Conservation Area (HRMCA) is a further concentration point within the region for recreational fishing. As this offshore fishing effort around the HRMCA was most probably focused onto tuna—which accounted for 63.4% of all interviewed fishers activities—the actual proportion of peninsular wide tuna fishing that was observed by CRAGS was potentially much higher than 23%.

For developing effort metrics, finding a representative site to observe may be important for precise tracking of trends. The tight rank correlation between paired regional bus‐route and CRAGS camera samples, suggest that effort at HRMCA was representative of regional temporal trends. Our results are also similar to other studies where sampling methods for recreational fisheries over broader scales, when compared to index trends of effort collected from point sources show strong correlations (Hartill et al., 2016).

CRAGS with its seascape scale of data capture observes actual effort on fishing grounds, rather than indirect metrics from movement past access points (Hartill et al., 2016; Smallwood, Pollock, Wise, Hall, & Gaughan, 2012; van Poorten & Brydle, 2018). This makes the choice of access points to monitor immaterial for trend collection and may be of particular interest for pelagic fisheries. While nonintuitive, recreational fishing for wide‐ranging migratory pelagic species is often spatially concentrated into small areas (Lynch, 2006), due to tight overlaps between ease of access by fishers and fish behavior related to bathymetry, migration, habitat, and prey availability (Lynch et al., 2004; Patterson, Evans, Carter, & Gunn, 2008).

Unlike spatial patterns, the intensity of coastal recreational temporal effort can be highly variable over time (Lynch, 2008, 2014; Wise, Telfer, Lai, Hall, & Jackson, 2012), though it is generally thought to follow predictable patterns relative to holiday and nonholiday periods (Jones & Pollock, 2012). Our results demonstrated these large fluctuations ranging from zero up to >400 hr of trailer boat effort for adjacent days. In combination with holiday periods, other sources of variability may also occur, due to individual or combinations of factors such as fishing club or competition activities and weather conditions. High‐frequency sampling can overcome these common temporal disjunctions in observed fishing effort, where by chance due to low numbers of samples logistically permissible with traditional roving or access point surveys, large errors in the estimation of effort can be introduced (Hartill et al., 2016; van Poorten & Brydle, 2018; van Poorten et al., 2015).

With multiple daily observations, abnormal episodes are more likely to be observed. For instance, a week‐long period of intense effort was recorded between 15 May 2015 and 27 May 2015 which was bracketed by two consecutive weekends where fishing competitions were held on the Tasman Peninsular. The normal sinusoid pattern of low weekday and high weekend effort was much less pronounced as the weekday lulls in effort were partially bridged by anglers continuing to fish. As bus‐route designs use day type as strata, with lower probabilities for sampling weekdays, and much fewer samples overall, this type of effect could easily be missed. While our bus‐route samples did overlap with the tuna fishing competitions, this was purely by chance. Such events, which result in large spikes of effort, can have marked impacts on local ecosystems and harvest estimates (McPhee et al., 2002; Monz, Pickering, & Hadwen, 2013) and, without prior knowledge, are more likely to be captured with monitoring at higher frequencies.

Methods for gathering data on coastal use, such as recreational fishing, moved away from direct approaches and toward off‐site methods such as telephone interviews (McCluskey & Lewison, 2008; Pollock et al., 1994) due to unsustainable costs from staffing and operations, particularly during periods of intense use, such as early mornings, weekends, and public holidays, which require overtime payments. However, sampling issues with off‐site methods are of particular concern for open‐access activities, such as unlicensed fisheries as there is no license database of contact phone numbers on which to base a sample frame. This is particularly so for niche recreational fisheries such as pelagic game fishing, as the fishers become increasingly dilute within the general population and hence are hard to access, particularly via off‐site methods such as telephone interviews (Griffiths et al., 2010).

Remote and autonomous deployment of sensors may therefore provide an alternative and cost‐effective method for long‐term monitoring, particularly for effort, across a range of applications. Our comparisons of matched pairs of data from two methods suggested that the CRAGS outputs were well correlated with regional effort trends. Our analysis of labor demonstrates that trying to understand this temporal variability with sampling via boat ramp surveys would be prohibitively expensive. Our CRAGS allows for a temporal effort metric, derived from actual observations of fishing, to be cheaply developed with sampling frequency at subdaily scales. This high‐frequency sampling approach was validated against our larger but much more costly bus‐route survey. Our analysis of labor required to both collect and process data, showed that CRAGS required considerably less resources to collect approximately nine times more frequent temporal data than the bus‐route method. Major labor savings were due to the ruggedized and autonomous capability of CRAGS, as only two field trips were required (deployment and retrieval took 7 FTE days), compared to the bus‐route's 16 field trips in a month which consumed 40 FTE days and included an additional 780 km of inter‐ramp road travel (Supporting Information Appendix S3). As it is weatherproof, autonomous and able to be programmed with a time delay setup CRAGS “fire and forget” nature both removes the need for observer to be physically located at the region to count activity (Edwards & Schindler, 2017) and work during overtime periods. The “blanking period” which stops and starts sampling—in this case overnight—also reduces battery and memory use. In combined, these technological advances have allowed long‐term CRAGS deployments across 4 years of 4–6 month rotations onto offshore islands and for several seasons in Antarctica to monitor seabirds (Lynch et al., 2017). The labor estimate for the bus route was conservative as it did not include overtime loading for the 6 weekend days of sampling or early morning commencement of a.m. samples.

The additional 3.81 FTE processing time required by the camera approach did not impact greatly on the overall labor budget when considered against fieldwork. The use of the Time Machine batch processing software to automatically produce the “big data” interactive movie was also a technological innovation that significantly reduced the labor of data handling compared to the manual opening of individual GigaPans used in previous studies (Lynch et al., 2015; Wood et al., 2016). In comparative terms, at the individual sample level to track the effort metric, each CRAGS sample required 2.4% of the labor of the bus‐route survey.

The development and application of image recognition software (machine learning) to detect, classify, and count boats automatically (Bousetouane & Morris, 2015) would further reduce postprocessing costs. The required frequency of sampling should also be considered on a case‐by‐case basis dependent on the research question as image collection can be somewhat open‐ended and result in unwieldy data sets that require subsampling to be cost‐effective (Parnell et al., 2010). In our case, the setting of ~5 images per day appeared to adequately describe the interdaily variation.

While our one autonomous camera provided a strong correlation with temporal trends in effort, compared to the regional survey, it did not provide relative spatial distributions of effort across the fishery. These distributions, however, can be predictable and highly concentrated (Lynch, 2006, 2014; Parnell et al., 2010), an observation that was also shown by our results, and much smaller levels of sampling effort would probably be required to resolve variation in spatial distributions compared to what is required to resolve the high temporal variations in effort. Understanding fine‐scale spatial distributions of recreational fishing effort is commonly achieved using aerial count, on‐water surveys, or vantage point surveys (Lynch, 2006; Smallwood et al., 2012), though other remote methods, such as high‐resolution satellite imagery (Fretwell, Scofield, & Phillips, 2017), may provide a cheaper solution. These types of wider surveys have also been suggested as highly compatible with point source metrics, such as remote camera systems, to allow for calibration or “up‐rating” for effort expansions to estimate overall effort within the fishery (Hartill et al., 2016).

The long distance (~6 km) between the HMRCA and the deployment location stretched the resolution limit of the current CRAGS setup, as we were unable to distinguish party size or activity type purely using the camera system. This is unlike previous CRAGS deployment, which had a high success rate of identifying party size/activity type at a shorter range of approximately 1.9 km (Wood et al., 2016). Thus, ground‐truthing information about the fishery was required for the system to work effectively. Autonomous camera system also do not collect creel (catch) data, but by allowing for cost‐effective collection of the highly variable temporal effort data they may allow for human resources to be spent elsewhere during survey periods (e.g., to undertake boat ramp interviews) (van Poorten & Brydle, 2018).

Our study was a small‐scale trial of new technology and while preliminary results are encouraging, validated seasonal effort estimations observed via CRAGS were outside the scope of this project. Our comparisons would hence have been improved by extending CRAGS deployments and with more comparisons to regional surveys. Similarly, using multiple CRAGS simultaneously would have better tested for differences in subregional patterns of temporal trends or concentration of in fishing effort, for instance, boats around the nearby Tasman Island.

In summary, the programmable sampling and data extraction offered by high‐resolution autonomous camera systems can allow for fine‐scale long‐term monitoring of fishing effort trends or potentially many other types of activities at seascape scales. More widespread adoption of high‐frequency sampling approaches for effort estimation would also help avoid collection of misrepresentative data by identifying effort anomalies (McCluskey & Lewison, 2008) such as the weekday continuation effect of raised effort between adjacent weekend fishing competition. By improving the information density over traditional survey methods, autonomous camera systems and other remote systems may provide a new wave of optimization for direct measurement of coastal human use, such as recreational fisheries, although complimentary methods are still required to determine spatial distributions, target species, and harvest.

## CONFLICT OF INTERESTS

The authors declare no conflict of interests.

## AUTHOR CONTRIBUTIONS

Mr Flynn helped design the study, led the fieldwork, undertook most of the analysis, and help write the manuscript. Dr Lynch help design the study, assisted in the fieldwork, provided advice on the analysis, and did some analysis, constructed figures, and after Mr Flynn contributed the most to the drafting of the manuscript. Dr Barrett helped design the study, assisted in some fieldwork, provided advice on analysis, and edited the manuscript. Mr Wong helped extensively with fieldwork, assisted with analysis, constructed figures, and helped write the manuscript. Ms Devine assisted with fieldwork and configuration of the equipment including the application of the big data movie software and handling of photographs, and also helped write the manuscript. Mr Hughes developed the electronics for the equipment, oversaw the build of gear, wrote the software to program the robot, and reviewed and edited the manuscript.

## DATA ACCESSIBILITY

Survey sampling schedules, data collection sheets, extrapolation procedures, and technical CRAGS specifications are all uploaded as online supporting information/appendix. Raw data from surveys and CRAGS: DOI: http://doi.org/10.5061/dryad.v10d218.

## RESEARCH ETHICS COMPLIANCE AND FUNDERS

Research abides by the Australian code of human research and experimentation (National Statement on Ethical Conduct in Human Research of 2007). Permission to conduct research on human participants was granted by the University of Tasmania: Social Science under reference: #H0014772—“Remote investigation of coastal area user group quantification and variation,” as well as from the CSIRO Social Science Human Research Ethics Committee under reference: 081/15—“Hawkesbury Region Project: Human Use Patterns.” Funding was by UTAS as Part of the IMAS Honours Project funding and through internal support from the Coasts Program.
