# Supplementary material for: Gigapixel big data movies provide cost‐effective seascape scale direct measurements of open‐access coastal human use such as recreational fisheries
Source: Ecol Evol. 2018 Aug 27;8(18):9372–83. doi: 10.1002/ece3.4301 (PMC6194282; doi:10.1002/ece3.4301)
Supplement: Supplementary file 1 [file ECE3-8-9372-s001.docx]

**Supplementary material**

**Appendix A**

**Camera Methods, Settings and CRAGS modifications**

Modifications include a new chips-set, firmware, power packs, housings, attachment systems and software. These additional features increase the flexibility of programing for sampling through blanking periods, flexible shooting schedules and delayed start times, weatherproofing and lowered power consumption for extended remote deployments.

CRAG modified chipsets was hardwired to the GigaPan keypad, therefore interfacing seamlessly with on-board hardware and firmware. A controller board with microUSB interface is inserted into a void space within the robot housing to override and control power to the robot, its mechanical servos and the synchronised camera. Unlike the factory GigaPan, the controller can be programmed (via microUSB interface) to power-down all systems between samples to a low power consumption of ~675uW (50 μA at 13.5V = 675uW). The latest firmware allows temporal sampling brackets to be blanked where samples are neither relevant or readable, such as night-time, avoiding superfluous data capture, reducing memory and battery demand and extending deployment length – all of these are features unavailable using a factory GigaPan unit. Each photographic image ‘tile’ within a sample’s mosaic was captured using a Canon 600D EOS Digital Single Reflex (DSLR) camera body (Chichibu, Saitama, Japan), through a Canon EFS 55–300mm lens with a Kenko 58 mm digital polarized filter onto a 128GB SanDisk Extreme memory card. The camera system was synchronized with our modified robotic GigaPan® EpicPRO camera mount using the 2.5mm jack and microUSB. This set-up was mounted inside a custom made water-proof housing which was developed by CSIRO and powered by an external water-proof battery pack (36,000mAh at 13.5v), all of this comprising one CRAG system. The system deployment was secured using an industrial tripod, mats, ropes and local rocks. Camera settings such as f-stop / aperture (10.0), ISO (400), shutter-speed (1/125s) and focus (2/3rds maximum target distance) were manually set, with white balance determined automatically for each tile.

**Appendix B**

 Calendar sampling schedules for ‘bus-route’ surveys made in May of 2015. Green = Weekdays and Yellow = Weekend or public holiday. Also, start site numbers 1, 2 and 3 correspond to Pirates Bay, Stuarts Bay and Fortescue Bay.

Bus-route field data collection sheet template used during interviews with trailer boat mariners. Note: “# ON BOARD correlates to party size for given vessel.

**Appendix C**

**Access Point (Bus-Route) Extrapolation**

The process for extrapolating effort from a traditional bus-route survey (Robson & Jones, 1989) was calculated through equation C.1. Our nominal boat

effort extrapolations were calculated from determining active boating time (minutes) based on counts of empty trailers, during an clerk’s wait time at a boat ramp. The active boating time ($e_{uv}$) ranged between 1 to 60 minutes, with a proportion of vessels entering or returning throughout the waiting period. Vessels were categorised as ‘in’ the water from the moment it was successfully launched; and conversely as ‘out’ of the water when successfully shackled to a trailer. Distance between sample sites, and hence overall route time ($T$) generally took ~1 hour to complete the ~39 km journey. Therefor route time was set at 4 hours for this study.

$$E_{ij}=\left( T\sum_{u=1}^{n} \frac{1}{w_{u}}\sum_{v=1}^{m} \frac{e_{uv}}{\pi_{u}} \right)$$

$$\times\hat{P}_{i}$$

Equation C.1

$E_{ij}$ = estimated boat-person effort for the day $i$ of day-type strata $j$

T = total route time (travel + waiting) to complete a full circuit of the route

$w_{u}$ = waiting time at the $u_{th}$ site

$e_{uv}$ = total time that the $v_{th}$ boat trailer was parked at the $u_{th}$ site within an clerk’s waiting time ($w_{i}$)

$\pi_{i}$ = sampling probability. The likelihood of the clerk’s sampling at the $u_{th}$ site.

The boating effort was extrapolated based on the monthly sampling frame. The total estimated effort (hours) ($Ê$) for a given month *k* was calculated with equation 8.

$$Ê_{k}= \sum\left( E_{ij1}+E_{ij2}\ldots E_{ijn} \right)$$

$Ê_{k}$ = estimated boat-person effort for month $k$

Equation C.2
